# Damage to the public health system caused by war-related looting or vandalism in the Tigray region of Northern Ethiopia

**DOI:** 10.3389/fpubh.2024.1271028

**Published:** 2024-04-05

**Authors:** Zenawi Hagos Gufue, Hiluf Kalayou Haftu, Yibrah Alemayehu, Etsay Weldekidan Tsegay, Meresa Berwo Mengesha, Berhe Dessalegn

**Affiliations:** ^1^Department of Public Health, College of Medicine and Health Sciences, Adigrat University, Adigrat, Ethiopia; ^2^Tigray Regional Health Bureau, Mekelle, Ethiopia; ^3^Department of Pharmacy, College of Medicine and Health Sciences, Adigrat University, Adigrat, Ethiopia; ^4^Department of Midwifery, College of Medicine and Health Sciences, Adigrat University, Adigrat, Ethiopia

**Keywords:** healthcare, costs, war, loss assessment, revitalization, Tigray, Ethiopia

## Abstract

**Background:**

The war that started on November 4, 2020, in the Tigray region of Northern Ethiopia severely affected the health sector. However, there is no available evidence to suggest the economic damage caused to the public health system because of war-related looting or vandalism. This study was aimed at estimating the cost of war-related looting or vandalism in Tigray’s public health system in Northern Ethiopia in 2021.

**Methods:**

A provider perspective, a mixed costing method, a retrospective cross-sectional approach, a 50% inflation rate, and a 50 Ethiopian birr equivalent to one United States dollar ($) for the money value were used. The data were analyzed using Microsoft Excel, taking into consideration the Sendai framework indicators.

**Results:**

The total economic cost of the war-related looting or vandalism in monetary terms was more than $3.78 billion, and the damage to the economic value in monetary terms was more than $2.31 billion. Meanwhile, the direct economic loss to the health system in monetary terms was more than $511 million. According to this assessment, 514 (80.6%) health posts, 153 (73.6%) health centers, 16 (80%) primary hospitals, 10 (83.3%) general hospitals, and 2 (100%) specialized hospitals were damaged and/or vandalized either fully or partially due to the war.

**Conclusion:**

This war seriously affected the public health sector in the Tigray region. The Federal Government of Ethiopia, the Ministry of Health of Ethiopia, the Tigrayan Government, the Tigray Regional Health Bureau, and the international community must make efforts to find resources for the revitalization of the damaged, plundered, and vandalized healthcare system.

## 1 Introduction

Tigray is located in the northern part of Ethiopia, the second-most populous country in Africa with an estimated population of 107,334,000. Whereas, the population of Tigray is estimated to be 5,838,000 (1). Along with the Ethiopian regions of Afar and Amhara, the region borders Sudan and Eritrea. It is the fifth-largest regional state in Ethiopia in terms of both population and area (2). The total war on November 4, 2020, between Ethiopian federal forces and its allies against Tigray forces arose in the name of law enforcement action (3).

After a bloody fight for a month, the federal government took control of Mekelle, the capital city of the Tigray region on November 28, 2021. The Tigrayan forces had changed the war technique and they continued guerrilla fighting outside of the urban territories. Despite the federal government seizing Mekelle, the Tigrayan forces recaptured again the capital city after 8 months of resistance on June 28, 2021 (3). Before the war broke out, Tigray had relatively better healthcare systems in Ethiopia, with over 720 health posts, 224 health centers, 24 primary hospitals, 14 general hospitals, 2 specialized hospitals, 250 ambulances, and more than 20,000 health workers (4).

The Tigray War caused a major humanitarian crisis, the collapse of the healthcare system, and the destruction of healthcare organizations (5). Tigray had over 90% of accessible public and private health facilities before the war (4). Since November 2020, millions of people have been internally displaced, causing a humanitarian crisis with food insecurity, destruction of medical facilities, and Tigrayan refugees fleeing Sudan (6). The Tigray Health Bureau and United Nations International Children’s Emergency Fund (UNICEF) assessed 263 health facilities, finding that 205 (77.9%) health centers and 24 (60%) hospitals were partially damaged. Of the 186 functional facilities, 40 (22%) were fully functional, 19% were partially functional, and 59% were non-functional. The major causes were medical supply, equipment, and skilled staff shortages (7).

The War significantly impacted healthcare delivery, with only 3.6% of health centers and 13.5% of hospitals, and none of the health posts were functional. The destruction varies by geographic location, with only 9.7% of health centers, 43.8% of general hospitals, and 21.7% of primary hospitals being fully functional. None of the facilities were operating owing to a lack of power, water supply, or ransacked spare parts (8). The number of people in need of emergency food assistance rose from less than 1 million to over 5.2 million, while pre-war rates of antenatal care, skilled delivery, postnatal care, and child vaccination were 64, 73, 63, and 73% (4).

Only 29 of the 230 health centers in Tigray were functional, and only 1% of facilities could provide comprehensive gender-based violence services (9). The public health sector’s economic value and direct losses from war-related health facilities can help finance Gross Domestic Product (GDP) losses for healthcare consumption. A case study in Eastern Ukraine revealed a lower-bound welfare loss for Donetsk residents ranging from 7.28 to 24.79% of lifetime income (10).

This conflict resulted in atrocities, massacres, and displaced populations. Over 91% of the population needed humanitarian assistance, with 150 hunger-related deaths and 350,000 people suffering from extreme food scarcity within 2 months after the Tigrayan forces recaptured most parts of the Tigray region (11, 12). However, few studies revealed that the war had a significant influence on Tigray’s healthcare system but lacked evidence of financial repercussions. This study sought to quantify direct economic losses and harm caused to the public health sector.

## 2 Methods and materials

### 2.1 Study area and period

This study was conducted in all accessible public health facilities and health offices in the Tigray region. Data were collected from July 2021 to November 2021, after the withdrawal of the Ethiopian, Eritrean, and Amhara forces from most parts of Tigray. Administratively, Tigray was divided into seven zones: the Central, Eastern, Mekelle, Northwestern, Southern, Southeastern, and Western zones. However, data was not collected from the Western Zone of Tigray and some districts of the eastern zone, the Northwestern Zones, due to security reasons.

### 2.2 Study design

A facility-based cross-sectional study design was used.

### 2.3 Study population

All health facilities and health offices were included, including health program managers, facility leaders, financial managers, pharmacists, healthcare professionals, health extension workers, and high-level experts. This study clearly defined the length of the study to be 1 year and 7 months to avoid recall biases and capture all costs related to war, based on the available literature, which is 1–2 years.

### 2.4 Sample size and sampling procedure

A total of 936 health facilities and administrative health offices from six zones of the Tigray region were included in the study. All health posts, health centers, primary hospitals, general hospitals, specialized hospitals, administrative health offices, health insurance agencies, research institutes, blood banks, and pharmaceutical supply companies were included in the study.

### 2.5 Costing, perspective, and cost type

This study used 17 cost estimation principles for global health services (13). The study estimated the economic value, direct economic losses, and productivity losses of the health system from a provider perspective. Validity was tested in a conflict-related damage assessment conducted by the Tigray Health Bureau and the World Health Organization. The median cost of input resources, including medicines, medical supplies, chemicals, and equipment, was obtained from the Ethiopian Pharmaceutical Supply Agency (EPSA) procurement list in 2020.

The study estimated economic damage, loss, productivity losses, and opportunity costs of the war in the health system using Ethiopian birr (ETB) and United States dollars (USD). Cost data agents include the Commercial Bank of Ethiopia (CBE), EPSA, the Bureau of Finance and Economic Development (BOFED), healthcare facility units, and primary data. The shadow prices for non-medical equipment were calculated for all health facilities and administrative offices. It used a 3% discount rate and 50% inflation rate equivalence for 2022 for medicines, reagents, chemicals, medical equipment, drugs, and non-medical items by adjusting capital costs and inflation. The median unit price was used, and the currency conversion rate was USD equivalent to 50 Ethiopian birr.

### 2.6 Sensitivity analysis

This study used the assumptions of depreciation cost analysis and replacement cost analysis (with and without depreciation and with and without inflation) to show the impact of the war on cost results and to determine the relationship between inputs and output results.

### 2.7 Operational definitions

Damage refers to the destruction, vandalism, or looting of health facilities, causing disruption or non-functionality. Damage includes the monetary value of destroyed assets that are repaired or replaced at the agreed replacement costs (14). Replacement cost: It is the cost an entity would pay to replace a destroyed asset at its pre-loss condition, not market value (14). Perspective: calculates costs by considering inputs and resources, addressing the economic loss of goods and services from war to full recovery and reconstruction (14).

Productivity: It is defined as the quantity of healthcare consumed and is quality-adjusted. In a competitive market, opportunity cost refers to the price of the most beneficial alternative resource. Health cost, or economic cost, is the benefit lost from not choosing an alternative. The opportunity cost of producing more units is reduced due to scarce resources. This study examines productivity loss in Tigray’s health system from 2019 to 2020, using the human capital approach and the 2020 gross earning salary. It uses the United Nations Development Programme (UNDP) Sendai framework indicators to assess the impact on GDP (15).

### 2.8 Inclusion and exclusion criteria

This study examined the direct economic damage, productivity loss, opportunity costs, and investment needs of healthcare workers affected by the war in the public health sector. It excluded private sector costs, health service delivery costs, disease morbidity costs, patient perspective costs, health information management system costs, and inaccessible areas due to security reasons.

### 2.9 Data quality assurance procedures

To collect high-quality data, 20 health system and medical institution data collectors and six experienced supervisors participated. They received instructions on war-related cost quantification goals, tools, and checklists that were customized for the local environment. Training enabled the necessary modifications to enhance the effectiveness of data collection.

### 2.10 Data management and analysis

The quality of the data was ascertained during the data entry and cleaning processes. The research team strictly supervised the data entry process during the entire period of the data entry process. Each quantification tool and cost center were strictly cleaned to avoid double-counting missed values so that unlikely values were rejected. Re-evaluation and judgment on the rejected responses were made, and appropriate actions were taken by a senior health economist. The data were adjusted for depreciation and inflation. Microsoft Excel was used for the 2021 EPSA quantification tool of medicines, medical supplies, medical equipment, laboratory reagents, and chemicals for annual forecasting and integrated damage assessment checklists, observations, and key informant interviews.

## 3 Results

A total of 638 health posts, 208 health centers, 20 primary hospitals, 12 general hospitals, 2 specialized hospitals, one regional health bureau, two blood banks, two health insurance agencies, one health research institute, two EPSA branches, and 48 woreda health offices from the six zones of Tigray were included in this cost estimation study. Five hundred fourteen (80.6%) health posts, 153 (73.6%) health centers, 16 (80%) primary hospitals, 10 (83.3%) general hospitals, and 2 (100%) specialized hospitals were damaged and/or vandalized either fully or partially because of the war.

The economic cost of the war was 189,176,643,518.98 birr ($3,783,532,870.38). The damaged economic value of this war was 115,873,981,221.14 birr ($2,317,479,624.42), and the direct economic loss of the health system was 25,591,665,235.89 birr ($511,833,304.72) (Table 1). The damaged economic value of health facilities and administrative offices was 115,873,981,220.24 birr ($ 2,317,479,624.40) (Table 2). The findings showed that the war-related cost of capital items, total fixed cost, was 61,483,981,221.00 birr (53.1%), and that of recurrent items, total variable cost, was 54,390,000,000.14 birr (46.9%) of the total economic value of damage (Table 3).

**Table 1:** Total estimated cost of war related looting or vandalism to the public health sector in the Tigray region of Northern Ethiopia, 2021.

Cost of war	In Ethiopian birr	In United States Dollar
Damage economic value	115,873,981,221.14	2,317,479,624.42
Direct economic loss	25,591,665,235.89	511,833,304.72
Productivity loss	1,280,149,767.71	25,602,995.35
Bill cost (sponsorship)	8,043,349,440.00	160,866,988.80
Opportunity cost	38,387,497,854.24	767,749,957.08
Total	189,176,643,518.98	3,783,532,870.38

**Table 2:** Damage economic value of health facilities and administrative offices in the Tigray region of Northern Ethiopia, 2021 (*n* = 936).

Facility type	# of facilities	In Ethiopian birr	In USD^1^
Health posts	638	19,654,270,468.80	393,085,409.38
Health centers	208	18,395,746,137.00	367,914,922.74
Primary hospitals	20	14,011,956,530.60	280,239,130.61
General hospitals	12	11,505,109,018.76	230,102,180.38
Comprehensive specialized hospitals	2	1,838,584,040.00	36,771,680.80
Blood banks	2	10,000,000.00	200,000.00
Woreda health offices and Health insurance agency	50	69,542,470.08	1,390,849.40
Tigray health bureau	1	13,090,155.00	261,803.10
Tigray health research institute	1	13,000,000.00	260,000.00
Ethiopian pharmaceutical supply agency	2	5,000,000.00	100,000.00
Infrastructures	–	50,357,682,400.00	1,007,153,648.00
Total	936	115,873,981,220.24	2,317,479,624.40

^1^USD, United States Dollar. 1 USD = 50 Ethiopian birr.

**Table 3:** The share of fixed and recurrent costs to the total damage economic value of the health system in the Tigray region of Northern Ethiopia, 2021.

Cost type	In Ethiopian birr	In United States Dollar
Total fixed cost	61,483,981,221.00	1,229,679,624.42
Total variable cost	54,390,000,000.14	1,087,800,000.00
Average cost	124,062,078.4	2,481,241.57
Total cost	115,873,981,221.14	2,317,479,624.42

1 USD = 50 Ethiopian birr.

The estimated cost of despoiled, forayed, ransacked, and pillaged medical equipment, drugs, medical supplies, and non-medical equipment and furniture for health posts was 21,531,789,140.40 birr ($ 430,635,782.81), and the total cost for health centeres was 18,395,746,133.44 birr ($367,914,922.67) (Table 4). The economic value of drugs, reagents, medical equipment, medical supplies, non-medical equipment, and furniture for primary hospitals was 14,011,956,530.60 birr ($ 280,239,130.61), while the economic damage value for general hospitals was estimated to be 11,505,109,019.16 birr ($ 230,102,180.38) (Table 5).

**Table 4:** Aggregated costs of damaged health centers and health posts in the Tigray region of Northern Ethiopia, 2021 (*n* = 846).

Items	Facility type	# of facilities	Unit cost (ETB^2^)	Total cost (ETB^2^)	Total cost (USD^1^)
Medical equipments	Health centers	208	4,416,540.33	918,640,388.64	18,372,807.77
	Health posts	638	2,945,765.00	1,879,398,070.00	37,587,961.40
Drugs and medical supplies	Health centers	208	3,874,943.00	805,988,144.00	16,119,762.88
	Health posts	638	340,158.20	217,020,931.6	4,340,418.63
Laboratory reagents	Health centers	208	5,075,500.00	1,055,704,000.00	21,114,080.00
	Health posts	638	2,492,250.00	1,590,055,500.00	31,801,110.00
Generator and/or solar light	Health centers	208	300,000.00	62,400,000.00	1,248,000.00
	Health posts	638	300,000.00	191,400,000.00	3,828,000.00
Ambulance	Health centers	208	5,000,000.00	1,040,000,000.00	20,800,000.00
Non-medical equipment and vehicles	Health centers	208	69,774,103.85	14,513,013,600.80	290,260,272.02
	Health posts	638	27,670,712.6	17,653,914,638.80	353,078,292.78
Total	Health centers			18,395,746,133.44	367,914,922.67
	Health posts			21,531,789,140.40	430,635,782.81

^1^USD, United States Dollar; ^2^ETB, Ethiopian birr; 1 USD = 50 Ethiopian birr.

**Table 5:** The damaged economic value of general and primary hospitals in the Tigray region of Northern Ethiopia, 2021 (*n* = 32).

Items	Facility type	# of facilities	Unit cost (ETB^2^)	Total cost (ETB^2^)	Total cost (USD^1^)
Medical equipments	General hospital	20	22,877,449.88	457,548,997.60	9,150,979.95
	Primary hospital	12	104,677,048.80	1,256,124,585.60	25,122,491.71
Drugs and medical supplies	General hospital	20	7,372,908.65	147,458,173.00	2,949,163.46
	Primary hospital	12	82,144,699.03	985,736,388.36	19,714,727.77
Laboratory reagents	General hospital	20	25,047,468.00	500,949,360.00	10,018,987.20
	Primary hospital	12	28,564,345.00	342,772,140.00	6,855,442.80
Generator	General hospital	20	300,000.00	6,000,000.00	120,000.00
	Primary hospital	12	500,000.00	6,000,000.00	120,000.00
Ambulance	General Hospital	20	5,000,000.00	100,000,000.00	2,000,000.00
	Primary hospital	12	5,000,000.00	60,000,000.00	1,200,000.00
Non-medical equipment and vehicles	General hospital	20	640,000,000	12,800,000,000	256,000,000
	Primary hospital	12	737,872,992.1	8,854,475,905.2	177,089,518.1
Total	General hospital			14,011,956,530.60	280,239,130.61
	Primary hospital			11,505,109,019.16	230,102,180.38

^1^USD, United States Dollar; ^2^ETB, Ethiopian birr; 1 USD = 50 Ethiopian birr.

The damaged economic value of drugs, reagents, medical equipment, medical supplies, non-medical equipment, and furniture for the comprehensive specialized referral hospitals was 1,838,584,040.04 birr ($36,771,680.80) (Table 6). Before the deadly war started in Tigray, the health system’s leadership and governance were intact, and all health administrative offices performed their daily activities well. The relationship between administrative health offices and the healthcare facilities was more robust than in any other region in Ethiopia. However, the bloody war compromised all the reform activities of the Tigray health system, and the estimated economic damage to the administrative health offices was 69,542,470.08 birr ($ 1,390,849.40).

**Table 6:** The damaged economic value of the comprehensive specialized hospitals in the Tigray region of Northern Ethiopia, 2021 (*n* = 2).

Items	# of facilities	Unit cost in (ETB^2^)	Total cost (ETB^2^)	Total cost (USD^1^)
Medical equipment	2	685,531,684.25	1,371,063,368.50	27,421,267.37
Drugs and medical supply	2	124,631,645.67	249,263,291.34	4,985,265.83
Non-medical equipment and vehicles	2	57,128,690.11	114,257,380.22	2,285,147.60
Ambulance	2	50,000,000.00	100,000,000.00	2,000,000.00
Generators	2	2,000,000.00	4,000,000.00	80,000.00
Total			1,838,584,040.04	36,771,680.80

^1^USD, United States Dollar; ^2^ETB, Ethiopian birr; 1 USD = 50 Ethiopian birr.

### 3.1 Sensitivity analysis

The study estimated the relationship between capital costs, recurrent costs, and affiliation of the total cost of the war. The depreciation of capital items and replacement cost assumption were the two assumptions used to show the input and output relationships for the study. Variable costs did not depreciate because their lifespan was less than 1 year.

### 3.2 Direct economic loss

Losses occur subsequently to the disaster in the health sector’s economic flows. These may appear at varying intervals after the disaster and can be more difficult to identify in a point study. Additional losses can occur from the time of the disaster until recovery and reconstruction. The direct economic loss of the public health sector was estimated about 57,573,652,404.35 birr ($ 1,046,793,680.0). The direct economic loss for 2021 was about 11,481,930,165.89 birr ($ 208,762,366.65) (Annex 1) and direct economic loss for 2022 was about 14,511,285,523.46 birr ($ 263,841,554.97) (Annex 2).

## 4 Discussion

The findings of this study demonstrate the economic cost of war-related looting, vandalism, and damage to the public health sector in the unoccupied parts of Tigray region of Northern Ethiopia. Cost consideration is very important for a rapid response in a time of crisis, and it helps to invest the maximum effort of leaders and managers to develop a strategic problem-solving activity at regional and national levels of the health system. In this study, we estimated the direct economic damage, direct economic loss, productivity loss, bill costs, and opportunity costs of the public health system.

The findings of this study showed that the economic cost of the war was 189,176,643,518.98 birr ($3,783,532,870.38), which was 10.5 times higher than the 2020 adjusted annual budget of the Tigray region (18 billion birr). It was 2.63 times higher than the total health spending of Ethiopia during 2016–17 (72.1 billion birr) and 3.81 times higher than the total health spending in 2013–2014 (49.6 billion birr) (16). The damage was 62.6% of Ethiopia’s GDP, it was 13.1 times higher than the global recommendation of less than 20% Out of Pocket Expenditure (OOPE) (14.4 billion birr).

The study showed that the OOPE was 8.5 times higher than the 2016/17 Ethiopian out-of-pocket expenses (22.32 billion birr) and 7.5 times higher than the share of external funding (25.2 billion birr). This study also showed that the cost of war-related looting or vandalism was 8.2 times higher than government health expenditure (23.04 billion birr) and 17.85 times higher than government *per capita* health expenditure (10.6 billion birr) in 2016/17 in Ethiopia (16).

The results of this study were comparable with those of the study conducted by the international monetary fund (IMF) to assess the economic consequences of conflict in Sub-Saharan Africa and show that, on average, annual growth in countries in intense conflict was about 2.5% lower and the cumulative impact on *per capita* GDP increased over time. Furthermore, conflicts pose significant strain on countries’ public finances, lowering revenue, rising military spending, and shifting resources away from development and social spending (17).

The findings of this study were also compared with those of previous studies conducted in Sri Lanka to estimate the economic costs of the war. A study conducted in Sri Lanka showed that the economic cost of the war was two times higher than the country’s GDP (18), which contrasted with the finding of this study by 0.63 times. This difference could be because this study focused only on the public health sector of the public health system. The difference could also be due to this study focusing only on one region of the country, and the study period may also be the cause of the difference because the length of time between the two studies was too long compared to the recommended less than 5 years.

The geographical location of the two regions could also be the reason for the difference because Tigray is located in the low-income sub-Saharan region of East Africa, but Sri Lanka is located in the middle-income countries of the Asian region, which brings in a GDP difference. A study conducted in Zurich, Switzerland, showed that healthcare costs were higher for patients from countries with violent conflict. The study showed that a person from a country with violent conflict had 94% higher odds of generating more costs (19).

The findings of this study also showed that the health system revenue lost for 2021 was 78.6% of the 2020 annual revenue, and the revenue lost for 2022 was four times that of the 2021 revenue loss, which made the hospitals not provide healthcare services to the needy community at an acceptable level of quality and at the least possible cost, in which people suffer from high out-of-pocket expenditures and catastrophic healthcare costs.

The findings of this study showed that they were 13.1 times higher than the global recommendation of less than 20% OOPE (14.4 billion birr) and 8.5 times higher than 2016/17 out-of-pocket expenses (22.32 billion birr), which is similar the study conducted in Donetsk Oblast, Ukraine. The study in Donetsk showed that the residents suffered at least 7.28 to 24.79% equivalent lifetime income loss compared to the pre-conflict period (10). The findings of this study showed a massive amount of war-related damage and loss (62.6%), which was consistent with the findings of a study conducted in El Salvador (20).

The study in El Salvador showed a huge economic deterioration, from a gross national product of 727 colones *per capita* to 665 in 10 years (14). It also showed that 514 (80.6%) Health Posts, 153 (73.6%) Health Centers, 16 (80.0%) primary hospitals, 10 (83.3%) General Hospitals, and 2 (10%) Specialized Hospitals were damaged and/or vandalized either fully or partially. The Tigray War involved a total war between the Tigray forces and affiliated forces, resulting in looting, vandalism, and destruction of health facilities. Soldiers had occupied every fifth facility, and few had ambulances (5). This study depicted the cost of war-related looting and vandalism in Tigray’s public health sector, focusing on health economists’ perspectives. This could aid bounce-back rehabilitation and help researchers develop medical strategies to reduce long-term war costs.

### 4.1 Strengths and limitations of the study

The study used global health cost methodology principles and validated tools, including UNDRR indicators. Even though it is axiomatic to face challenges in any scientific research studies the following were among the common challenges we face during our study, lack of transportation access, black out of communication materials, and limited financial support. However, it does not show the total cost of the war on Tigray’s health system, as it continued after the study period. In addition, it does not include the impact of health service delivery or direct OOPE. However, this study’s cost quantification for the public health sector only shows the total cost of war and siege, without considering private health sector costs, damaged utilities, data and patient charts, Western Tigray, and additional health system investments.

## 5 Conclusion

The Tigray region’s health system faced severe damage due to the brutal and rough-and-tumble nature of the war. As a result, the delivery of healthcare services was severely hampered, and numerous health facilities were looted, damaged, and destroyed. Consequently, severe acute malnutrition, maternal, infant, and child mortality rates are frequently observed. In the region, shortages of medication and medical supplies caused problems for patients with chronic illnesses, such as cancer, diabetes mellitus, and hypertension, and the prevalence of communicable infections rose sharply. To restore the damaged, looted, and vandalized health sector in Tigray, the Tigray health bureau, the government of Tigray region, the government of Ethiopia, and the international community needed to pay special attention to mobilizing healthcare resources.

## Data availability statement

The original contributions presented in the study are included in the article/Supplementary material, further inquiries can be directed to the corresponding author.

## Ethics statement

To obtain clearance for the study, the research team went through an ethical review process with Mekelle University’s institutional review board, referencing the number MU-IRB/1906/2021. Each participant received a written informed consent form, confirming that they were aware of the study’s purpose and agreed to participate. The research team took great care to keep all collected data strictly confidential. To ensure anonymity, all collected data was stored and analyzed with no personal identifiers. The research study was carried out following the guidelines of the 1964 Declaration of Helsinki, ensuring that the participants’ dignity, rights, and welfare were fully protected.

## Author contributions

ZG: Conceptualization, Formal analysis, Supervision, Writing – original draft, Writing – review & editing. HH: Conceptualization, Data curation, Formal analysis, Funding acquisition, Investigation, Methodology, Project administration, Resources, Software, Supervision, Validation, Visualization, Writing – original draft, Writing – review & editing. YA: Investigation, Methodology, Project administration, Supervision, Writing – original draft, Writing – review & editing. ET: Formal analysis, Software, Writing – review & editing, Methodology, Validation. MM: Data curation, Formal analysis, Validation, Writing – review & editing. BD: Conceptualization, Data curation, Formal analysis, Software, Supervision, Writing – review & editing.
