# Supplementary material for: Damage to the public health system caused by war-related looting or vandalism in the Tigray region of Northern Ethiopia
Source: Front Public Health. 2024 Apr 5;12:1271028. doi: 10.3389/fpubh.2024.1271028 (PMC11026641; doi:10.3389/fpubh.2024.1271028)
Supplement: Supplementary file 1 [file Data_Sheet_1.PDF]

Annex 1:Direct Economic Loss of the public health sector in the Tigray region of Northern Ethiopia, 2021.

Conflict related direct Economic loss, of the health system in Tigray in 2021.				
**Direct economic loss in 2021.**				
**Lost revenues**	**Economic loss**		**Opportunity cost**	
	**In Ethiopian birr**	**In USD**	**In Ethiopian birr**	**In USD**
Adjusted budget	1,828,785,382.44	33,250,643.32	426,106,994.11	7,747,399.89
IRR+ fee waiver	227,352,641.06	4,133,684.38	52,973,165.37	963,148.46
DEL for exempted services	1,810,911,891.96	32,925,670.76	421,942,470.83	7,671,681.29
PW revenue	10,969,083	199,437.87	2,555,796.32	46,469.02
Health insurance	133,648,177	2,429,966.86	31,140,025.34	566,182.28
Humanitarian responses	5,200,000,000.00	94,545,454.55	1,211,600,000.00	22,029,090.91
Specialized hospitals loss	1,207,274,594.64	21,950,447.18	281,294,980.55	5,114,454.19
**Total**	**10,418,941,770**	**156,509,634.15**	**2,427,613,432.52**	**458,409,215.58**
**Drug, reagent, diagnostic, cost of program services loss in 2021.**				
Program services	**In Ethiopian birr**	**In USD**	**In Ethiopian birr**	**In USD**
Annual budget for HIV and STI	214,515,474.84	3,900,281.36	49,982,105.64	908,765.56
Annual budget for TBL	38,899,205.10	707,258.27	9,063,514.79	164,791.18
Annual budget for malaria	82,559,433.60	1,501,080.61	19,236,348.03	349,751.78
Annual budget for nutrition	43,903,981.08	798,254.20	10,229,627.59	185,993.23
Annual budget for vaccine and vaccine accessories	173,897,338.95	3,161,769.80	40,518,079.98	736,692.36
Annual budget for COVID-19	63,151,989.48	1,148,217.99	14,714,413.55	267,534.79
Annual budget for family planning and contraceptives	148,739,481.80	2,704,354.21	34,656,299.26	630,114.53
**Total**	**765,666,904.85**	**13,921,216.45**	**178,400,388.83**	**3,243,643.43**
**Administrative cost of program services loss in 2021.**				
**Program services**	**In Ethiopian birr**	**In USD**	**In Ethiopian birr**	**In USD**
Annual budget for HIV and STI	52,001,033.58	945,473.34	12,116,240.82	220,295.29
Annual budget for TBL	16,200,000.00	294,545.45	3,774,600.00	68,629.09
Annual budget for malaria	36,000,000.00	654,545.45	8,388,000.00	152,509.09
Annual budget for nutrition	7,867,501.20	143,045.48	1,833,127.78	33,329.60
Administrative cost for MNCH services	85,323,295.40	1,551,332.64	19,880,327.83	361,460.51
Annual budget for COVID-19	99,929,660.40	1,816,902.92	23,283,610.87	423,338.38
**Total**	**297,321,490.58**	**5,405,845.28**	**69,275,907.31**	**1,259,561.95**
**Over all direct economic loss summary in 2021.**				
**Lost revenues**	**In Ethiopian birr**	**In USD**	**In Ethiopian birr**	**In USD**
DEL for budget, IR, PW, HI, military expenditure	10,418,941,770.46	189,435,304.92	2,427,613,432.52	44,138,426.05
DEL drugs, reagents for program services	765,666,904.85	13,921,216.45	178,400,388.83	3,243,643.43
DEL for program administrative costs	297,321,490.58	5,405,845.28	69,275,907.31	1,259,561.95
**Total**	**11,481,930,165.89**	**208,762,366.65**	**2,675,289,728.65**	**48,641,631.43**

Coronavirus disease 2019, DEL: Direct Economic Loss, HI: Health Insurance, HIV: Human immune deficiency virus, IRR: Internal Rate of Revenue, MNCH: Maternal, neonatal and child health, STI: Sexually Transmitted Disease, TBL: TB and Leprosy, USD: United States Dollar.

Annex 2: Direct economic loss of the public health sector in the Tigray region of Northern Ethiopia, 2022.

War related direct economic loss of the health sector in the Tigray region, under health bureau in 2022.				
**Lost revenues**	**Economic loss**		**Opportunity cost**	
	**In Ethiopian birr**	**In USD**	**In Ethiopian birr**	**In USD**
Adjusted budget	2,106,257,844.59	38,295,597.17	490,758,077.79	8,922,874.14
IRR+ fee waiver	261,847,720.52	4,760,867.65	61,010,518.88	1,109,282.16
Exempted services	9,054,559,459.80	164,628,353.	2,109,712,354.13	38,358,406.44
PW revenue	12,633,367	229,697.58	2,943,574.52	53,519.54
Specialized hospitals loss	1,207,274,595	21,950,447.18	281,294,980.55	5,114,454.19
Health insurance	307,390,808	5,588,923.78	71,622,058.29	1,302,219.24
Humanitarian responses	498,333,333.33	9,060,606.06	116,111,666.67	2,111,121.21
**Total**	**13,448,297,128**	**244,514,493.24**	**3,133,453,230.83**	**56,971,876.92**
**Drug, reagent, diagnostic, cost of program services loss in 2022.**				
**Program services**	**In Ethiopian birr**	**In USD**	**In Ethiopian birr**	**In USD**
Annual budget for HIV and STI	214,515,474.84	3,900,281.36	49,982,105.64	908,765.56
Annual budget for TBL	38,899,205.10	707,258.27	9,063,514.79	164,791.18
Annual budget for malaria	82,559,433.60	1,501,080.61	19,236,348.03	349,751.78
Annual budget for nutrition	43,903,981.08	798,254.20	10,229,627.59	185,993.23
Annual budget for vaccine and vaccine accessories	173,897,338.95	3,161,769.80	40,518,079.98	736,692.36
Annual budget for COVID-19	63,151,989.48	1,148,217.99	14,714,413.55	267,534.79
Annual budget for family planning and contraceptives	148,739,481.80	2,704,354.21	34,656,299.26	630,114.53
**Total**	**765,666,904.85**	**13,921,216.45**	**178,400,388.83**	**3,243,643.43**
**Administrative cost of program services loss in 2022.**				
**Program services**	**In Ethiopian birr**	**In USD**	**In Ethiopian birr**	**In USD**
Annual budget for HIV and STI	52,001,033.58	945,473.34	12,116,240.82	220,295.29
Annual budget for TBL	16,200,000.00	294,545.45	3,774,600.00	68,629.09
Annual budget for malaria	36,000,000.00	654,545.45	8,388,000.00	152,509.09
Annual budget for nutrition	7,867,501.20	143,045.48	1,833,127.78	33,329.60
Administrative cost for MNCH program services	85,323,295.40	1,551,332.64	19,880,327.83	361,460.51
Annual budget for COVID-19	99,929,660.40	1,816,902.92	23,283,610.87	423,338.38
**Total**	**297,321,490.58**	**5,405,845.28**	**69,275,907.31**	**1,259,561.95**
**Over all direct economic loss summary.**				
**Lost revenues**	**In Ethiopian birr**	**In USD**	**In Ethiopian birr**	**In USD**
DEL for budget, IRR, PW, HI, and humanitarian	13,448,297,128.03	244,514,493.24	3,133,453,230.83	56,971,876.92
DEL drugs, reagents for program service	765,666,904.85	13,921,216.45	178,400,388.83	3,243,643.43
DEL for program administrative costs	297,321,490.58	5,405,845.28	69,275,907.31	1,259,561.95
**Total**	**14,511,285,523.46**	**263,841,554.97**	**3,381,129,526.97**	**61,475,082.31**

Coronavirus disease 2019, DEL: Direct Economic Loss, HI: Health Insurance, HIV: Human immune deficiency virus, IRR: Internal Rate of Revenue, MNCH: Maternal, neonatal and child health, STI: Sexually Transmitted Disease, TBL: TB and Leprosy, USD: United States Dollar.
